# Intelligent maneuver strategy for hypersonic vehicles in three-player pursuit-evasion games via deep reinforcement learning

**DOI:** 10.3389/fnins.2024.1362303

**Published:** 2024-02-14

**Authors:** Tian Yan, Zijian Jiang, Tong Li, Mengjing Gao, Can Liu

**Affiliations:** Unmanned System Research Institute, Northwestern Polytechnical University, Xi’an, China

**Keywords:** hypersonic vehicles, deep neural network, reinforcement learning, pursuit-evasion game, three players, intelligent maneuver strategy

## Abstract

Aiming at the rapid development of anti-hypersonic collaborative interception technology, this paper designs an intelligent maneuver strategy of hypersonic vehicles (HV) based on deep reinforcement learning (DRL) to evade the collaborative interception by two interceptors. Under the meticulously designed collaborative interception strategy, the uncertainty and difficulty of evasion are significantly increased and the opportunity for maneuvers is further compressed. This paper, accordingly, selects the twin delayed deep deterministic gradient (TD3) strategy acting on the continuous action space and makes targeted improvements combining deep neural networks to grasp the maneuver strategy and achieve successful evasion. Focusing on the time-coordinated interception strategy of two interceptors, the three-player pursuit and evasion (PE) problem is modeled as the Markov decision process, and the double training strategy is proposed to juggle both interceptors. In reward functions of the training process, the energy saving factor is set to achieve the trade-off between miss distance and energy consumption. In addition, the regression neural network is introduced into the deep neural network of TD3 to enhance intelligent maneuver strategies’ generalization. Finally, numerical simulations are conducted to verify that the improved TD3 algorithm can effectively evade the collaborative interception of two interceptors under tough situations, and the improvements of the algorithm in terms of convergence speed, generalization, and energy-saving effect are verified.

## 1 Introduction

With the development of anti-near-space technology, especially the progress of cooperative interception technology against the hypersonic vehicle (HV) (Ding et al., 2022), the survival space of HV encountering two interceptors has been greatly compressed. Its high-speed advantage will not lead the HV to achieve successful evasion once again (Liu et al., 2023). Accordingly, it is essential to investigate the problem that one HV faces with two interceptors and design the relevant game maneuver strategy to achieve successful evasion.

The HV’s evasion for two interceptors is essentially a special three-player pursuit-evasion game problem and the three-player pursuit-evasion game problem, nowadays, has been widely studied. Weintraub et al. (2020) described the pursuit-evasion game with the application in aerospace in detail and Pachter and Wasz (2019) focused on the field of vessels. Zhang et al. (2022), Casini and Garulli (2022), and Fang et al. (2020) studied the three-player game in which the speeds of the two pursuers are greater than, equal to, and less than that of one evader, respectively. Nath and Ghose (2022) and Zhang and Zha (2018) conducted pursuit-evasion game research under certain objectives or constraints in the two-dimensional plane. The differential game strategy in optimal control (Fuchs et al., 2018; Szots et al., 2021; Yan et al., 2021; Zhang et al., 2023) was utilized to solve the three-player pursuit-evasion game. Wang et al. (2020), Wan et al. (2021), and Hamidoglu (2023) each applied different intelligent algorithms to achieve evasion, respectively. Unlike the above references, the particularity of the hypersonic three-player pursuit-evasion problem is the vehicle characteristics of both sides as well as the special pursuit-evasion confrontation scenarios (Liu et al., 2022). The pursuer (interceptor) can offset the speed difference of a few Mach and magnify the overload disadvantage of the evader (HV) by constructing the head-on situation where the pursuer and the evader fly toward each other in opposite directions. In addition, when multiple pursuers form cooperative interception under the sensible interception strategy, the maneuvering space of the evader will be further compressed and the difficulty of HV’s successful evasion will be greatly increased, unlike the two-evader/one-pursuer problem (Liang et al., 2022) or the target-defense-attack problem (Sinha et al., 2022). In conclusion, the hypersonic three-player pursuit-evasion game is a highly dynamic and strongly adversarial pursuit-evasion game problem in complex situations, which is extremely difficult for the evader under reasonable confrontation scenarios and interception strategy.

Therefore, the examination of the hypersonic three-player pursuit-evasion game is based on creating logical pursuit-evasion game situations as well as creating cooperative interception techniques for pursuers. The two most notable studies of HV evasion versus two interceptors in recent years (Yan et al., 2020; Shen et al., 2022) both noted that the hypersonic pursuit-evasion problem must be considered in head-on situations. To guarantee the difficulty of HV evasion, Yan et al. (2020) further designed the confrontation scenarios belonging to head-on situations that several Successive Pursuers came from the Same Direction (SPSD) and proposed the hierarchical cooperative interception strategy to form the coordinated interception with layered interferences in time and space. By carefully designing the spacing ΔX between two interceptors, efforts were made to ensure that at least one interceptor intercepted the pursuer. Therefore, when investigating the hypersonic pursuit-evasion game, this paper chooses to apply the hierarchical cooperative interception strategy (Yan et al., 2020), and further expands and constructs the attack and defense confrontation model based on the SPSD scenario.

Furthermore, the optimum control approach was employed by Yan et al. (2020) and Shen et al. (2022) to create HV maneuver overload orders. Shen et al. (2022) chose to transform the HV’s trajectory optimization problem of evading two interceptors into a nonconvex optimal control problem and solved it by the interior point method, while Yan et al. (2020) derived an analytical expression for the evasion command satisfying certain constraints. These two strategies had high requirements on the onboard computer resources, computation time, and real-time access to the information of interceptors, which are difficult to achieve in practical applications. Therefore, it is imperative to use more potent intelligent algorithms to capture maneuver time and resolve the hypersonic three-player pursuit-evasion puzzle.

Deep reinforcement learning (DRL), an emerging intelligence algorithm, has found widespread use in hypersonic vehicles. The algorithm obtains the optimal policy by continuous trial-and-error and feedback learning through constant interaction with the environment, and it has the perceptual capability of deep learning (DL) and the decision-making capability of reinforcement learning (RL), allowing end-to-end perception and decision in high-dimensional state-action space (Matsuo et al., 2022). A large body of literature utilized DRL in the HV pursuit-evasion problem. Gaudet et al. (2020) and Gaudet et al. (2021) developed a guidance law for an outer atmospheric interception based on proximal policy optimization (PPO) and meta-learning. The trust region policy optimization (TRPO) algorithm was proposed to generate an interception guidance law (Chen et al., 2023). With an emphasis on the terminal evasion scenario, Qiu et al. (2022), based on DRL, developed a maneuver evasion guidance method that took into account both guidance accuracy and evasion capabilities. In a different study (Jiang et al., 2022), the problem was reformulated as a Markov decision process (MDP), and an Actor-Critic (AC) framework-based DRL algorithm was used to solve it to suggest the anti-interception guiding law. To intercept the moving target, Li et al. (2022) somewhat enhanced the reinforcement learning algorithm. The ideal attitude-tracking problem for HVs during the reentry phase (Zhao et al., 2022) was solved using the RL algorithm. Bao C. et al. (2023) produced the three-dimensional (3D) trajectory of the HV during the glide phase using the RL algorithm and deep neural network (DNN). The autonomous optimum trajectory planning technique for the HV was designed using the deep deterministic policy gradient (DDPG) algorithm (Bao C. Y. et al., 2023) minimizing the trajectory terminal position errors. Gao et al. (2023) and Guo et al. (2023) both applied the two delay deep deterministic (TD3) policy gradient algorithm to solve the HV’s one-to-one pursuit-evasion game problem in the head-on situation and a series of improvements were made (Guo et al., 2023) to expand the application scenarios and enhance the performance of the algorithm. It is worth mentioning that, while DRL algorithms have been widely used to solve HV pursuit-evasion problems, they are all confined to how one HV evades one interceptor and how several interceptors block the HV. As far as the authors know, no literature has employed the DRL algorithm to address how the HV evades two interceptors in challenging scenarios, which is due to the HV’s inability to elude two interceptors easily. Cooperative interception methods in unfavorable scenarios increase the randomness and uncertainty in the highly dynamic game process, making it challenging to successfully train the agent. Furthermore, various key performance factors in HV pursuit-evasion, such as generalizability and energy consumption, should be prioritized.

As a result, this study picks the TD3 algorithm applied to continuous action space and performs targeted changes to develop the intelligent maneuver strategy to handle the hypersonic three-player pursuit-evasion problem. Firstly, the three-player attack and defense confrontation model of a hypersonic vehicle encountering two interceptors is established. Secondly, the three-player pursuit-evasion problem is modeled as a Markov decision process and the double training strategy is proposed to take into account both interceptors and guarantee the whole training success. At the same time, the reward functions are carefully designed to compromise the terminal miss distance and the energy consumption during the evasion process by an adjustable energy-saving factor. In addition, the structure of the deep neural network of the TD3 algorithm is improved and the regression network is introduced to enhance the generalization of the intelligent maneuver strategy.

The advantages of the proposed intelligent maneuvering approach over classical methods (Yan et al., 2020; Shen et al., 2022) are as follows. Compared to the ballistic optimization approach (Shen et al., 2022), the proposed strategy based on the DRL algorithm is created by continuous interactions between both sides throughout the adversarial game rather than unilateral design. Furthermore, in this article, the beginning conditions are constructed as more difficult close-range frontal situations in which unilateral ballistic planning based on mass maneuvering fails to achieve successful evasion. Meanwhile, the proposed method continually investigates each maneuver strategy through interaction and eventually converges on the superior solution rather than the conservative solution, which is superior to the traditional maneuver strategy (Yan et al., 2020). Furthermore, the proposed strategy does not take up too many resources of the ballistic computer and does not need to capture the information of the pursuers at any time during the pursuit-evasion process.

The main innovations of this paper are as follows.

1. To the best of the authors’ knowledge, the proposed strategy is the first intelligent maneuver strategy based on the DRL algorithm for solving the hypersonic three-player pursuit-evasion problem under tough situations.
2. In this paper, the relationship between the off-target amount and energy consumption of HV is fully considered, and the energy-saving factor is set in reward functions to quantitatively regulate the above two important indexes.
3. This paper improves the generalization of the algorithm, and the regression network is designed in the deep neural network of the TD3 algorithm to improve network structure to apply to complex confrontation situations.

The remaining research is organized as follows. Section 2 describes the confrontation scenarios and the model of the pursuit-evasion game problem for HV evading two interceptors. In Section 3, the TD3 algorithm is introduced and targeted improvement strategies are proposed. The proposed strategy is verified by numerical simulation in Section 4. Conclusions are presented in Section 5.

## 2 Model and problem

In this section, the attack and defense confrontation scenarios in which the HV encounters two interceptors are designed. Then, the hypersonic three-player pursuit-evasion problem is modeled under these scenarios and the corresponding detailed formulations are given.

### 2.1 Pursuit-evasion confrontation scenarios

When designing pursuit-evasion confrontation scenarios between HV and interceptors, interceptors must constitute head-on situations against the HV regardless of the number of interceptors. Only in these situations can the interceptor successfully engage the HV (*Remark 1*). For the three-player pursuit-evasion game with two pursuers, the pursuers’ interception strategy is crucial. From the perspective of anticipating the enemy, this paper chooses the hierarchical cooperative interception strategy (*Remark 2*), which constitutes the SPSD pursuit-evasion confrontation situations for HV. In addition, considering the HV maneuvering characteristics, the attack and defense confrontation scenarios are simplified to a two-dimensional plane (*Assumption 1*).

**Figure 1:**
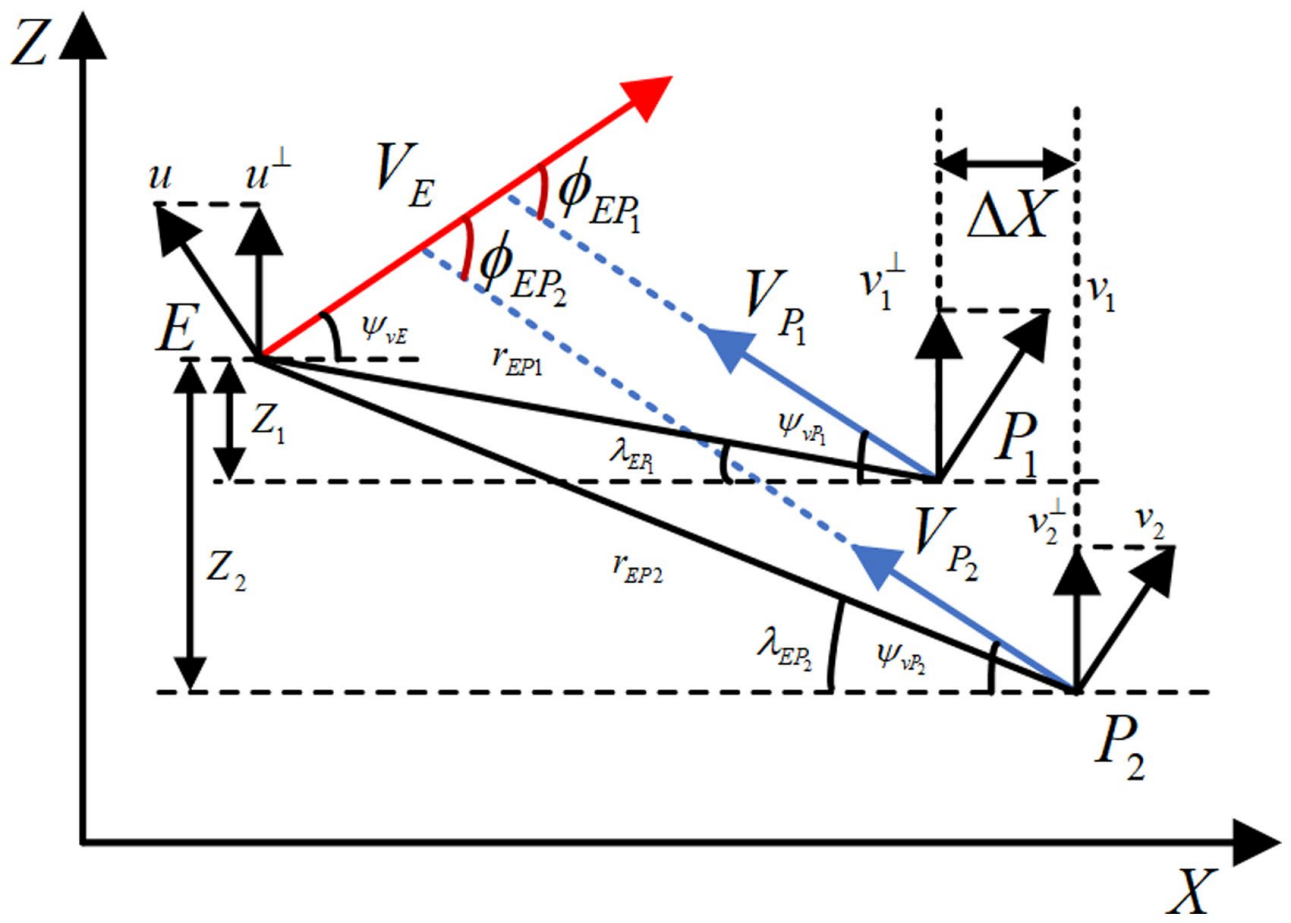
X-Z plane adversarial geometry of three-player.

The relative motion diagram is shown as Figure 1 and the relative geometric kinematics equations for the HV encountering two interceptors are given as follows:


(1) $$\left\{\begin{array}{l} {\dot{r}}_{EP_{i}}=-V_{E}\cos\left(\psi_{VE}-\lambda_{EP_{i}}\right)+V_{P_{i}}\cos\left(\psi_{VP_{i}}+\lambda_{EP_{i}}\right)\\ {\dot{\lambda }}_{EP_{i}}=\left(V_{E}\sin\left(\psi_{VE}-\lambda_{EP_{i}}\right)-V_{P_{i}}\sin\left(\psi_{VP_{i}}+\lambda_{EP_{i}}\right)\right)/r_{EP_{i}}\\ {\ddot{r}}_{EP_{i}}=u\sin\left(\psi_{VE}-\lambda_{EP_{i}}\right)+v_{i}\sin\left(\psi_{VP_{i}}+\lambda_{EP_{i}}\right)+r_{EP_{i}}{\dot{\lambda }}_{EP_{i}}^{2}\\ {\ddot{\lambda }}_{E_{i}}=\left(u\cos\left(\psi_{VE}-\lambda_{EP_{i}}\right)+v_{i}\cos\left(\psi_{VP_{i}}+\lambda_{EP_{i}}\right)-2{\dot{r}}_{EP_{i}}{\dot{\lambda }}_{EP_{i}}\right)/r_{EP_{i}}\\ \psi_{VE}=u/V_{E}\\ \psi_{VP_{i}}=-v_{i}/V_{P_{i}} \end{array}\right.$$

where $E,P_{i}\;\left(i=1\text{,}2\right)$ represent the evader (HV) and two pursuers (interceptors) respectively, which are regarded as mass points. $r_{EP_{i}}\left(i=1\text{,}2\right)$ indicate the relative distances between the evader and two pursuers, respectively. $\lambda_{EP_{i}}\left(i=1\text{,}2\right)$ are the line-of-sight angles between the evader and the two pursuers, respectively. $u,v_{i}\left(i=1\text{,}2\right)$ are the overloads of the HV and interceptors, respectively. $V_{j}\left(j=E\text{,}P_{i}\right)$ denote the velocities of three aircraft respectively, which are considered to be constant during the game process (*Assumption 2*), and $\psi_{Vj}\left(j=E\text{,}P_{i}\right)$ are the ballistic declination angles of the three aircraft. As shown in Figure 1, the magnitudes of the velocity intersection angle $\phi_{EP_{i}}\left(i=1\text{,}2\right)$ are set within a range of smaller values to ensure the head-on situation, and the spacing of the pursuers ΔX is established to guarantee that interceptors can form the cooperative interception.

Considering the vehicle characteristics, the respective dynamics and kinematics equations for HV and interceptors can be given by:


(2) $$\{\begin{array}{l} \frac{\text{d}V_{j}}{\text{d}t}=g\left(n_{xj}-\sin\theta_{j}\right)\\ \frac{\text{d}\theta_{j}}{\text{d}t}=\frac{g}{V_{j}}\left(n_{yj}-\cos\theta_{j}\right)\\ \frac{\text{d}\psi_{Vj}}{\text{d}t}=-\frac{g}{V_{j}\cos\theta_{j}}n_{zj} \end{array}$$

(3) $$\{\begin{array}{l} \frac{\text{d}x_{j}}{\text{d}t}=V_{j}\cos\theta_{j}\cos\psi_{Vj}\\ \frac{\text{d}y_{j}}{\text{d}t}=V_{j}\sin\theta_{j}\\ \frac{\text{d}z_{j}}{\text{d}t}=-V_{j}\cos\theta_{j}\sin\psi_{Vj} \end{array}$$

where the subscripts $j=E,P_{i}\;\left(i=1\text{,}2\right)$ denote the engaged aircraft of the pursuit-evasion in both parts. $\theta_{j}\left(j=E\text{,}P_{i}\right)$ indicate the ballistic inclination angles of aircraft, respectively. x,y,z are the coordinates of vehicles in three directions, and n is the vehicle overload, $n_{zE}=u,n_{zP_{i}}=v_{i}\left(i=1\text{,}2\right)$ .

Considering the small-angle hypothesis, the linear differential equation for the hypersonic three-player pursuit-evasion game can be expressed as:


(4) $$\begin{array}{l} \overset{\cdot }{x}=Ax+B_{u}u_{c}+B_{v,1}v_{c,1}+B_{v,2}v_{c,2}\\ u^{⊥}=c_{P}^{⊤}x+d_{P}u_{c}\\ v_{i}^{⊥}=c_{E,i}^{⊤}x+d_{E,i}v_{c,i} \end{array}$$

where $u^{⊥}=u\cos\psi_{VE}$ , $v_{i}^{⊥}=v_{i}\cos\psi_{VP_{i}}\left(i=1\text{,}2\right)$ . $u_{c},v_{c,i}\left(i=1\text{,}2\right)$ are the overload commands for HV and two interceptors, $u,v_{i}\left(i=1\text{,}2\right)$ denote the corresponding overload responses as well. The state variable can be selected as $x=\left[z_{1}\text{,}\overset{\cdot }{z_{1}}\text{,}z_{2}\text{,}\overset{\cdot }{z_{2}}\text{,}x_{E}\text{,}x_{P1}\text{,}x_{P2}\right]$ . Among them, z is the bias of both pursuit-evasion parties in the longitudinal direction. $z_{1}=z_{E}-z_{P1}$ , $z_{2}=z_{E}-z_{P2}$ . $x_{E},x_{P1},x_{P2}$ are the state variable of evader and pursuers.


(5) A=0 1 0 0 01×nE01×nP101×nP20 0 0 0 cE⊤−cP1⊤01×nP20 0 0 1 01×nE01×nP101×nP20 0 0 0 cE⊤01×nP1−cP2⊤0nE×10nE×10nE×10nE×1AE0nE×nP10nE×nP20nP1×10nP1×10nP1×10nP1×10nP1×nEAP10nP1×nP20nP2×10nP2×10nP2×10nP2×10nP2×nE0nP2×nP1AP2

(6) Bu=0dE0dEbE0nP1×10nP2×1,Bv,1=0−dP10 00nE×1bP10nP2×1,Bv,2=000−dP20nE×10nP1×1bP2

The coefficients in the above equations are shown as:


(7) $$\{\begin{array}{l} A_{E}=-\frac{1}{\tau_{E}},b_{E}=\frac{1}{\tau_{E}}\\ A_{P_{i}}=-\frac{1}{\tau_{P_{i}}},b_{P_{i}}=\frac{1}{\tau_{P_{i}}}\\ c_{E}=\cos\psi_{VE0},c_{P_{i}}=-\cos\psi_{VP_{i}0}\\ d_{E}=d_{P_{i}}=0 \end{array}$$

where $\tau_{j}\left(j=E\text{,}P_{i}\right)$ are the time constants of the first-order control system of both pursuit-evasion vehicles, and $\psi_{Vj0}\left(j=E\text{,}P_{i}\right)$ are the initial ballistic declination angles of the three vehicles. In the article, Eqs. 1–3 are applied for the subsequent verification in numerical simulations, while Eqs. 4–7 are used to derive and describe the physical quantities used.

And Longitudinal deviations are given as scalar, that is:


(8) $$z_{1}=c_{1}^{⊺}x,z_{2}=c_{2}^{⊺}x$$

where $\begin{array}{l} c_{1}^{⊺}=\left[1\;\;\;0\;\;\;0\;\;\;0\;\;\;0_{1\times n_{E}}\;0_{1\times n_{P_{1}}}0_{1\times n_{P_{2}}}\right],\\ c_{2}^{⊺}=\left[0\;\;\;0\;\;\;1\;\;\;0\;\;\;0_{1\times n_{E}}\;0_{1\times n_{P_{1}}}0_{1\times n_{P_{2}}}\right] \end{array}$ .

The guidance command $v_{c,i}$ for the ith pursuer, $i\left(i=1\text{,}2\right)$ , is denoted as:


(9) $$v_{c,i}=F_{i}\left(x\text{,}t\right)=N_{i}V_{r_{i}}{\dot{\lambda }}_{EP_{i}}+\frac{u}{2}$$

where the augment proportional guidance (APN) law is chosen to be the intercept guidance law. $N_{i}\left(i=1\text{,}2\right)$ are the guidance coefficients of interceptors, and $V_{r_{i}}\left(i=1\text{,}2\right)$ are the respective approach velocity.

**Figure 2:**
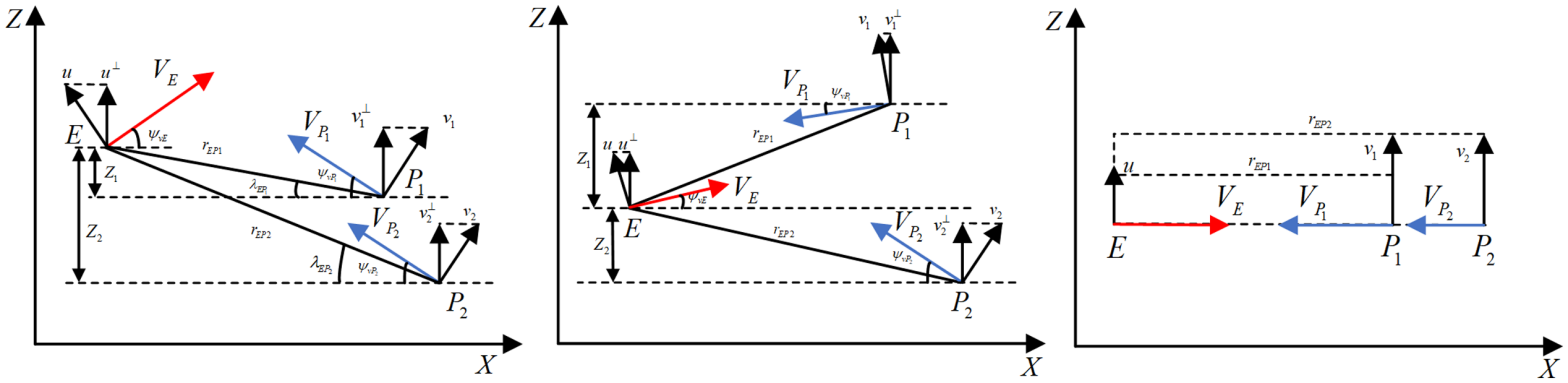
Expanded SPSD adversarial scenarios.

In addition, based on the classic SPSD scenario in Figure 1, this paper further expands the initial adversarial situations by shifting the initial parameters under a certain magnitude, which are categorized into scenarios of three types according to the changes in the line-of-sight angle $\lambda_{EP_{1}}\lambda_{EP_{2}}>0;\lambda_{EP_{1}}\lambda_{EP_{2}}<0;\;\;\lambda_{EP_{1}}=\lambda_{EP_{2}}=0$ , as shown in Figure 2.

The above initial situations in Figure 2, which are highly likely to occur in practical engineering applications, form the extended SPSD adversarial scenarios. Since the hypersonic three-player pursuit-evasion game is a highly dynamic and strongly stochastic problem, the above three adversarial scenarios should be considered simultaneously when designing the HV’s maneuver strategy. The extended SPSD confrontation scenarios proposed above have certain research significance.

### 2.2 Hypersonic three-player pursuit-evasion game problem

In the hypersonic three-player pursuit-evasion problem, the HV’s evasion is considered successful if the minimum relative distances between the HV and both interceptors are greater than the critical miss distance, that is:


(10) $$r_{EP_{1}}\left(t_{f_{1}}\right)>\delta_{1}\cap r_{EP_{2}}\left(t_{f_{2}}\right)>\delta_{2}$$

where $t_{f_{i}}\left(i=1\text{,}2\right)$ denote the terminal time when HV meets two interceptors. $\delta_{i}\left(i=1\text{,}2\right)$ indicate the critical miss distance of the ith interceptor.

In addition, considering the characteristics of HV itself, it is necessary to set process constraints for HV overload, namely:


(11) $$|u\left(t\right)|\leq u_{H\max}$$

Moreover, considering the requirement for subsequent flights or striking targets after HV evasion, it is essential to consider maneuver energy consumption during the evasion process and optimize HV evasion energy consumption under the premise of success evasion, that is:


(12) $$\overset{t_{f}}{\underset{t_{0}}{\int }}u^{2}\left(t\right)\text{d}t$$

In summary, the hypersonic three-player pursuit-evasion problem can be formulated as *Problem 1*:

*Problem 1*: Based on the hypersonic three-player pursuit-evasion game model (Eq. 4) and the guidance laws of interceptors (Eq. 9), the maneuver strategy is generated based on the intelligent algorithm, which can achieve the regulation of maneuver energy consumption (Eq. 12) while satisfying the terminal off-target quantity constraint (Eq. 10) and the process control constraint (Eq. 11).

*Remark 1*: The head-on situation is a prerequisite for the investigation of the hypersonic pursuit-evasion game. Since under non-head-on situations, the HV can easily escape utilizing its speed advantage. On the contrary, under the head-on situation, HV’s speed advantage is canceled out and interceptors utilize larger overloads than HV’s to achieve successful interception.

*Remark 2*: The hierarchical cooperative interception strategy is to construct the interception scenario in which multiple consecutive interceptors, coming from the same direction with appropriate intervals (Yan et al., 2020), create hierarchical interference in time and space. The core of the cooperative interception lies in the design of interceptor spacing ΔX . If the spacing ΔX is set appropriately, when evading the first interceptor, HV must consider how to evade the second interceptor, so as to achieve the interception effect of 1 + 1 > 2.

*Assumption 1*: The hypersonic three-player pursuit-evasion game is investigated under a two-dimensional plane.

*Remark 3*: Influenced by the inherent characteristics of HV engines, HV tends to evade interceptors by lateral maneuvers on the horizontal plane. Therefore, assuming that interceptors and HV are engaged in a pursuit-evasion game at the same altitude, the confrontation scenarios can be simplified to the X-Z two-dimensional plane.

*Assumption 2*: Both the interceptors and the HV keep maneuvering at a constant velocity.

*Remark 4*: The speed newly produced by longitudinal overload $n_{x}$ is negligible compared with the far supersonic speed of flight. Compared to longitudinal overload $n_{x}$ , lateral overload $n_{z}$ is the main factor in achieving maneuver evasion, which is perpendicular to the direction of velocity and does not change the magnitude of the velocity.

## 3 Method

The problem of hypersonic pursuit-evasion is a hot spot in the current research on hypersonic vehicles, whose difficulty lies in how to seize the maneuver timing to achieve successful evasion in the highly dynamic game confrontation. When facing the cooperative interception of two interceptors, the space and timing of the HV maneuver are further compressed. It is one effective solution to obtain reasonable maneuver strategies in complex game confrontation scenarios through deep reinforcement learning, which can solve the sequential decision-making problem by gradually improving the maneuver strategies based on the reward feedback in the interaction with the environment (Bai et al., 2023).

**Figure 3:**
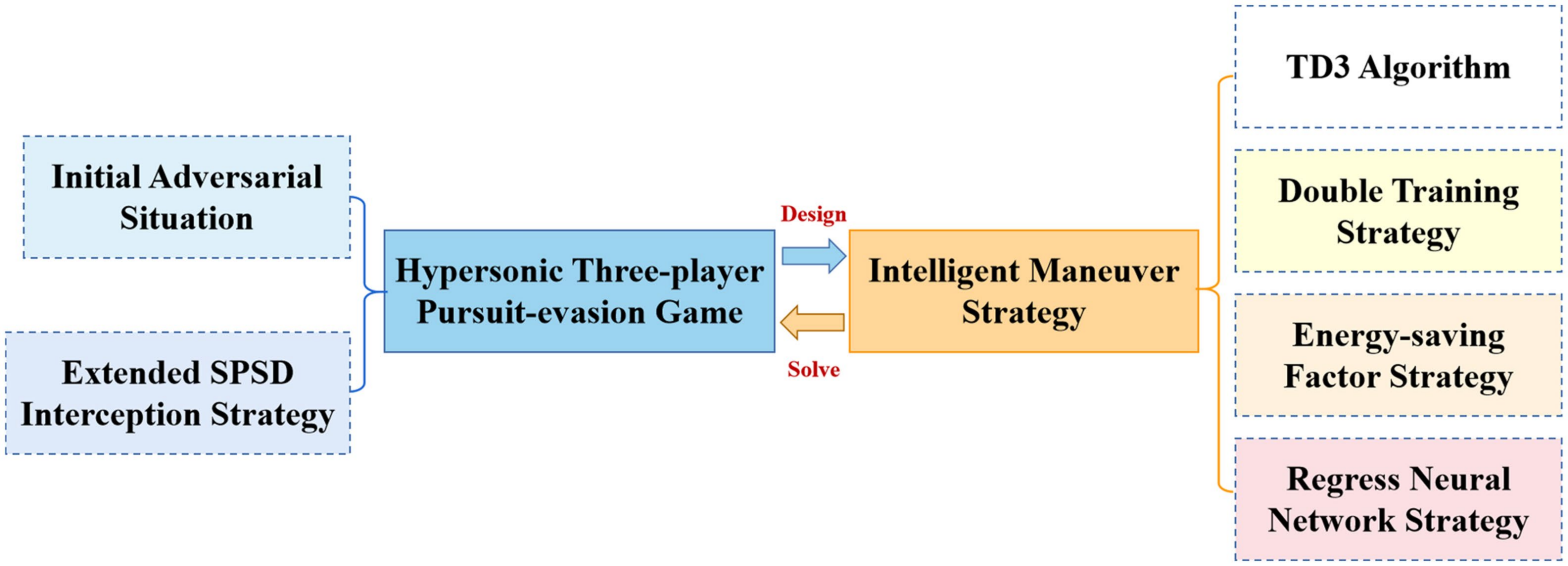
Block diagram of intelligent maneuver strategy.

In this paper, based on the two delay deep determined policy gradient (TD3) algorithm in deep reinforcement learning, the intelligent maneuver strategy is targeted to be designed with three improvement strategies to solve the hypersonic three-player pursuit-evasion game in Figure 3. The double training strategy is proposed to reduce the training difficulty and improve the convergence of the algorithm for cooperative interception strategies in unfavorable situations. The regression network is newly added to the deep neural network structure of the TD3 algorithm to improve the generalization. The reward functions are carefully designed and the energy-saving factor is set to quantitatively regulate the amount of off-target and energy consumption.

**Figure 4:**
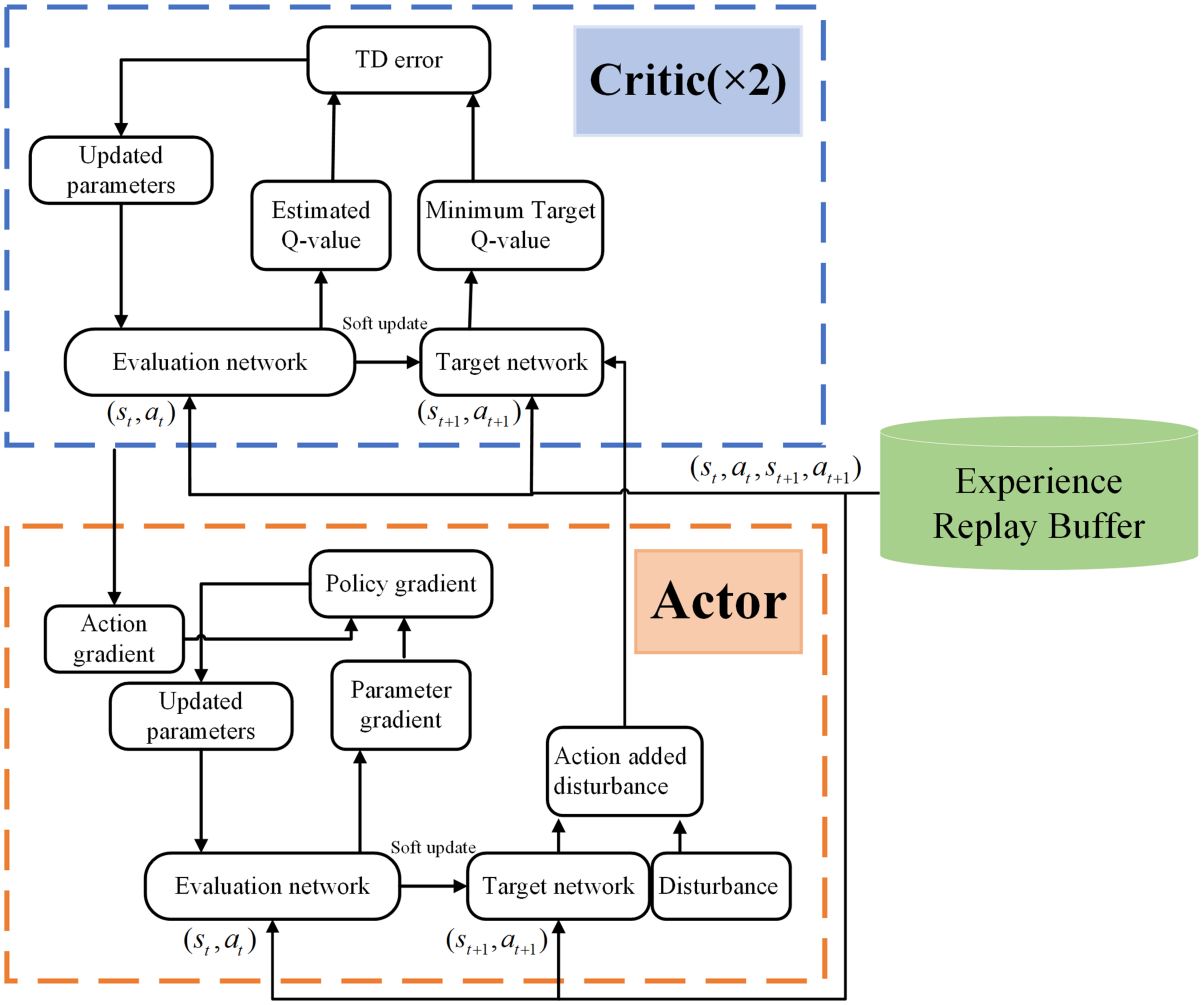
TD3 algorithm framework.

### 3.1 The TD3 algorithm

The hypersonic three-player pursuit-evasion game can be modeled as a Markov decision process (MDP) before solving it using the DRL algorithm.

The MDP can be represented by the quintuple $\left\{S\text{,}A\text{,}P\text{,}R\text{,}\gamma \right\}$ consisting of state S , action A , reward R , transfer function P , and discount factor γ . The specific MDP update formulas are given as follows:


(13) $$s^{\prime }=p\left(s^{\prime }\text{|,}s\text{|,}a\right)=P_{r}\left(S_{t}=s\text{|,}S_{t-1}=s\text{|,}A_{t-1}=a\right)$$

(14) $$r_{t}=R\left[S_{t}=s\text{,}A_{t}=a\right]$$

(15) $$P_{ss'}^{a}=P\left[S_{t+1}=s^{\prime }\text{|,}S_{t}=s\text{|,}A_{t}=a\right]$$

In the DRL algorithm, the agent’s goal is to learn the optimal policy function $\pi \left(a\text{|}s\right)$ , to maximize the cumulative mathematical expectation of the agent’s reward, namely the Q function of the state-action function.


(16) $$\pi \left(a\text{|}s\right)=P_{r}\left(A_{t}=a\text{|}S_{t-1}=s\right)$$

(17) $$\{\begin{array}{l} G_{t}=\underset{j}{\sum }\gamma^{k}r_{t+j+1}\\ Q\left(s_{t}\text{,}a_{t}\right)=E_{\kappa \sim \pi }\left(G_{t}\right) \end{array}$$

where $\gamma \in \left[0\text{,}1\right]$ is the discount factor, which measures the size of the future reward in the cumulative reward in the current state, and κ is the future state trajectory obtained by sampling according to the strategy π . Since the future state trajectory is unknown, only an estimate of the Q function $\overset{\wedge }{Q}$ can be computed.

As shown in Eq. 17, whether the Q value can be accurately estimated or not has a great impact on the performance of the strategy π . Regardless of too large or too small $Q\left(s\text{,}a\right)$ , the policy π will not be able to output the optimal action. The TD3 algorithm can evaluate the value accurately, based on the Actor-Critic (AC) framework, which mainly provides a parallel structure for actions and evaluations at the same time to deal with high-dimensional state space and continuous action space.

Figure 4 shows the structural framework of the TD3 algorithm. The Actor network $C_{\phi }$ outputs the current action $a_{t}$ according to the current state $s_{t}$ , and the Actor target network $C_{\phi '}$ outputs the target action $\tilde{a_{t}}$ according to the next state $s_{t+1}$ . The Critic network $Q_{\theta_{i}}$ calculates the $Q_{\theta_{i}}\left(s_{t}\text{,}a_{t}\right)$ value in the state $s_{t}$ and the action $a_{t}$ , and the Critic target network $Q_{\theta_{i}^{'}}$ calculates the target Q value according to the next state $s_{t+1}$ and the target action $\tilde{a_{t}}$ . ϕ and $\theta_{i}$ are the parameters of the Actor network and Critic network, respectively, as well as $\phi^{\prime }$ and $\theta_{i}^{'}$ are the parameters of the Actor target network and Critic target network respectively, i=1,2 . To improve the over-estimation problem of the DDPG algorithm, the smaller Q value of the two Critic target networks is selected as the target value $y_{t}$ , when updating the parameters of the Critic network.


(18) $$\Delta \theta_{i}=\nabla_{\theta_{i}}{\left(y_{t}-Q_{\theta_{i}}\left(s_{t},a_{t}\right)\right)}^{2}$$

(19) $$y_{t}=r_{t}+\gamma \min_{i=1,2}Q_{\theta_{i}^{'}}\left(s_{t+1},{\tilde{\alpha }}_{t}\right)$$

(20) $${\tilde{\alpha }}_{t}=C_{\phi^{\prime }}\left(s_{t+1}\right)+\varepsilon^{\prime }$$

where ε is the random noise obeying a truncated normal distribution $clip\left(N\left(0\text{,}\sigma \right)\text{,}-c\text{,}c\right),c>0$ . The parameters of the Actor network and the Actor target network are updated as follows:


(21) $$\Delta \phi =\nabla_{\phi }Q_{\theta_{1}}\left(s_{t},C_{\phi }\left(s_{t}\right)\right)$$

(22) $$\theta_{i}^{\prime }\leftarrow \tau \theta_{i}+\left(1-\tau \right)\theta_{i}^{\prime }$$

(23) $$\phi^{\prime }\leftarrow \tau \phi +\left(1-\tau \right)\phi^{\prime }$$

where τ⩽1 .

The TD3 algorithm, as a DRL algorithm applied to high-dimensional state space and continuous action space, effectively alleviates the over-estimation problem of DDPG, and its convergence speed and stability are better than the same type of DRL algorithms, which can be utilized for solving the hypersonic three-player pursuit-evasion problem. But considering the difficulty of the hypersonic three-player pursuit-evasion game, it is needed to make targeted improvements on its basis.

### 3.2 The double training strategy

For the hypersonic three-player pursuit-evasion problem, considering its characteristics of high dynamics and strong confrontation under a multi-body game, if the TD3 algorithm is applied directly, it is difficult to ensure the stability of the algorithm and converge to the optimal strategy during training. To solve the above problem, consulting relevant literature (Xu et al., 2019; Zhong et al., 2022), this paper proposes the joint planning idea of “expert guidance + intelligent algorithm optimization.”

The expert guidance refers to the double training strategy leading to successful agent training that the intelligent maneuver strategy can be successfully generated through two training with sequential order. The reason for the success of training based on the double training strategy is that the HV evading hierarchical cooperative interception strategy of two interceptors has been modeled as a Markov decision process in Section 3.1, whose essence is that the current state is only related to the state of the previous moment, but not related to the state of the state before the previous moment.


(24) $$p\left(s_{i+1}\mathrm{|,}s_{i},a_{i}\right)=p\left(s_{i+1}|s_{i},a_{i},\cdots ,s_{0},a_{0}\right)$$

Considering the time sequence between the respective meeting between two interceptors and HV under the specific interception strategy, HV’s successful evasion of the second interceptor must be based on the successful evasion of the first interceptor. In other words, HV’s whole maneuver strategy to evade the cooperative interception of the two interceptors $\pi_{whole}\left(a\text{|}s\right)$ is included in the maneuver strategy to evade the first interceptor $\pi_{1}\left(a\text{|}s\right)$ . And the strategy $\pi_{whole}\left(a\text{|}s\right)$ is the same as the maneuver strategy to evade the second interceptor $\pi_{2}\left(a\text{|}s\right)$ which is a subset of the maneuver strategy to evade the first interceptor $\pi_{1}\left(a\text{|}s\right)$ .


(25) $$\pi_{whole}\left(a\text{|}s\right)=\pi_{2}\left(a\text{|}s\right)\subset \pi_{1}\left(a\text{|}s\right)$$

Therefore, the intelligent maneuver strategy can be trained firstly aimed at the first interceptor nearby and retrained on its basis for the second interceptor at a later time. The complex and highly dynamic multi-player pursuit-evasion problem is transformed into several one-on-one pursuit-evasion problems depending on the character of the problem itself, which effectively reduces the blindness of the algorithm in the early stage of exploration.

**Figure 5:**
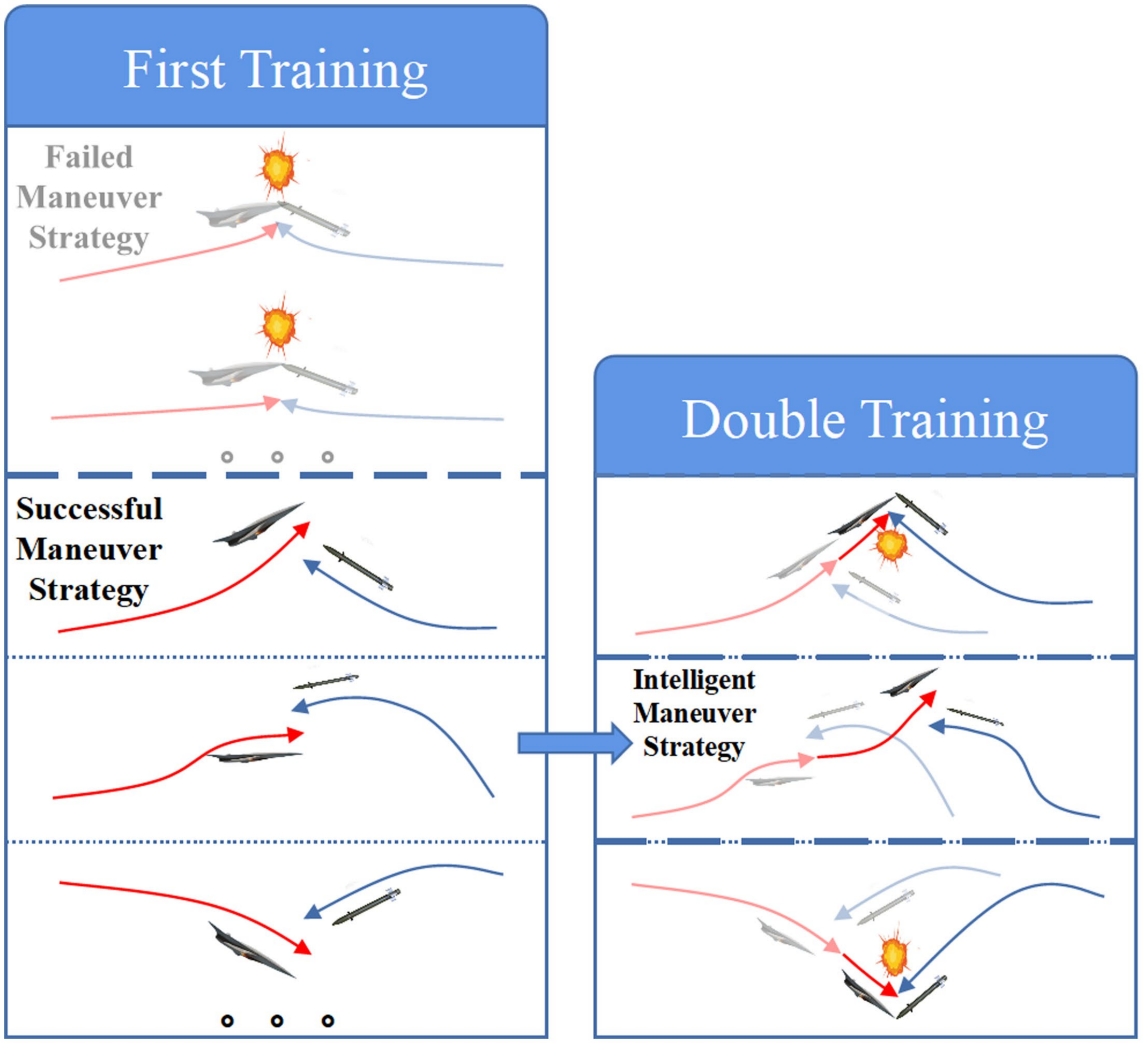
Double training strategy.

The intelligent algorithm optimization means that the TD3 algorithm is used in both training to train and converge to the optimal solution under the current game confrontation. The schematic diagram of the proposed double training strategy based on “expert guidance + intelligent algorithm optimization” is shown in Figure 5.

As shown in Figure 5, based on the double training strategy, the successful maneuver strategy against the first interceptor can be obtained through the first training, and the intelligent evasion strategy against cooperative interception can be generated by further optimization based on the first training.

In addition, the traditional one-shot training method is not discarded, and the specific sampling allocation is as follows:


(26) $$N_{batch}=w_{1}N_{double}+w_{2}N_{single}$$

where the samples $N_{double}$ generated by the double training strategy and the samples $N_{single}$ obtained from single training are assigned by weights $w_{1}$ and $w_{2}$ , which are both input into the experience pool to ensure the stability of the algorithm.

### 3.3 The energy-saving factor strategy

In this section, this paper focuses on analyzing the relationship between the off-target amount of terminal evasion and the energy consumption of the evasion process in the hypersonic three-player pursuit-evasion game. To realize the quantitative regulation of the two parts, the reward functions are carefully designed and the concept of energy saving factor is newly introduced.

Evasion off-target amount and maneuver energy consumption are the two most important indicators in the HV pursuit-evasion game, in which the off-target amount reflects the terminal performance and the energy consumption is the indicator in the process. By comparing the size of the off-target amount and critical miss distance, it can directly reflect whether HV evasion is successful or not, while the energy consumption in the process will affect HV’s subsequent flights and striking targets. In addition, off-target amount and energy consumption are contradictory in the whole pursuit-evasion flight of HV that the expectation of increasing off-target amount often requires larger maneuvering overload consuming more energy while saving energy consumption will inevitably lead to the decrease of off-target amount. Shen et al. (2022) and Yan et al. (2020) both modeled the overload energy consumption in the performance index and minimized the energy consumption under the premise of successful evasion. Gao et al. (2023) pursued a larger off-target amount, and the overload only needed to satisfy the constraints. Guo et al. (2023) realized the adaptive adjustment between off-target amount and energy consumption through the design of reward functions. However, considering the complex environment of HV in the flight process and the unknown situation it may have to face in the future, the overload energy consumption and terminal off-target amount in the HV evasion should be quantitatively adjusted. For this reason, this paper sets an energy-saving factor in reward functions, and changing the size of the energy-saving factor can quantitatively regulate the above two major indexes.

Reward function design is the focus and difficulty in reinforcement learning, which directly determines whether the training can be successful and whether the final strategy can be obtained or not. The reward functions can also be divided into the process reward function and the terminal reward function. Among them, the terminal reward directly determines whether the training is successful or not, while the process reward will guide the agent to obtain the key actions in different states through interaction with the environment leading to the success of the training. In addition, considering the intelligent maneuver strategy generated through the double training strategy in Section 3.2, there should be two sets of reward functions for the two incoming interceptors in front of and behind each other.

The reward functions for the first interceptor are as follows:


(27) $$R_{1}=r_{11}+r_{12}+r_{13}+r_{14}$$

(28) $$\{\begin{array}{l} r_{11}=c_{1}\ln\left({\overset{\cdot }{\lambda }}_{EP_{1}}\right)+c_{2}\\ r_{12}=c_{3}e^{-\frac{r_{EP_{1}}}{100}}\\ r_{13}=c_{4}\log_{2}\left(r_{EP_{1}}\left(t_{f1}\right)-\left(\delta_{1}-1\right)\right)\\ r_{14}=\{\begin{array}{l} -10,failure\\ 10,success \end{array} \end{array}$$

where $r_{11},r_{12}$ belong to the process rewards, while $r_{13},r_{14}$ belong to the terminal rewards. The process reward focuses on the line-of-sight angle and relative distance during the pursuit-evasion game to guide HV to deviate from the first interceptor, and the terminal reward is set up about the terminal off-target amount in addition to the rewards or penalties brought by evasion success or failure.

The reward functions for the second interceptor are as follows:


(29) $$R_{2}=r_{21}+r_{22}+r_{23}+r_{24}$$

(30) $$\{\begin{array}{l} r_{21}=c_{5}\left(1-E\right)\left(-\left(\frac{1}{r_{EP_{2}}}-\frac{1}{r_{EP_{2}0}}\right)+\mathrm{||}{\overset{\cdot }{\lambda }}_{EP_{2}}\mathrm{||}\right)\\ r_{22}=c_{6}Ee^{-\left(u-\left(u_{\max}-1\right)\right)}\\ r_{23}=c_{7}t\\ r_{24}=\{\begin{array}{l} 10\left(r_{EP_{2}}\left(t_{f2}\right)-1\right),success\\ -10,failure \end{array} \end{array}$$

The reward functions for the second interceptor are also divided into process and terminal rewards, and an energy-saving factor is introduced in the reward functions for the second interceptor as well. The energy-saving factor E is used to set the training tendency to aim for a larger amount of off-target or lower energy consumption. The essence of the energy-saving factor strategy is to assign weights ( 1−E and E ) to two performance indicators (off-target amount and energy consumption) to influence the training tendency of the TD3 algorithm. Considering the characteristics of the terminal off-target amount and energy consumption, the sum of the weighting factors is set to 1. In addition, the energy-saving factor strategy is only meaningful under successful HV evasion, therefore, the terminal reward functions are also designed for the second interceptor to ensure successful evasion.

### 3.4 The regress network strategy

In this section, the deep neural network structure of the TD3 algorithm is analyzed and improved, and the generalization of the algorithm is enhanced by introducing the regression network.

Insufficient generalization is a common problem in DRL algorithms, the training scenario and parameters are relatively fixed, which makes the agent obtained from training perform well under the feature points, however, when the application scenario changes, it is difficult for the agent to generate the optimal strategy. And the algorithm suffers from the defects of reduced effectiveness and insufficient generalization.

The TD3 agent trained based on the above improvement strategies can indeed generate intelligent maneuvering strategies to successfully circumvent the cooperative interception under the feature points. However, this paper expands the scenarios based on the classic SPSD situation. Considering the randomness of the problem, although the initial postures of the two sides are biased to a certain extent during the TD3 training, the agent will still fail to evade in individual initial situations. Therefore, to improve the generalization of the algorithm, this paper improves the network structure of the TD3 algorithm by introducing the regression network into the original deep neural network.

**Figure 6:**
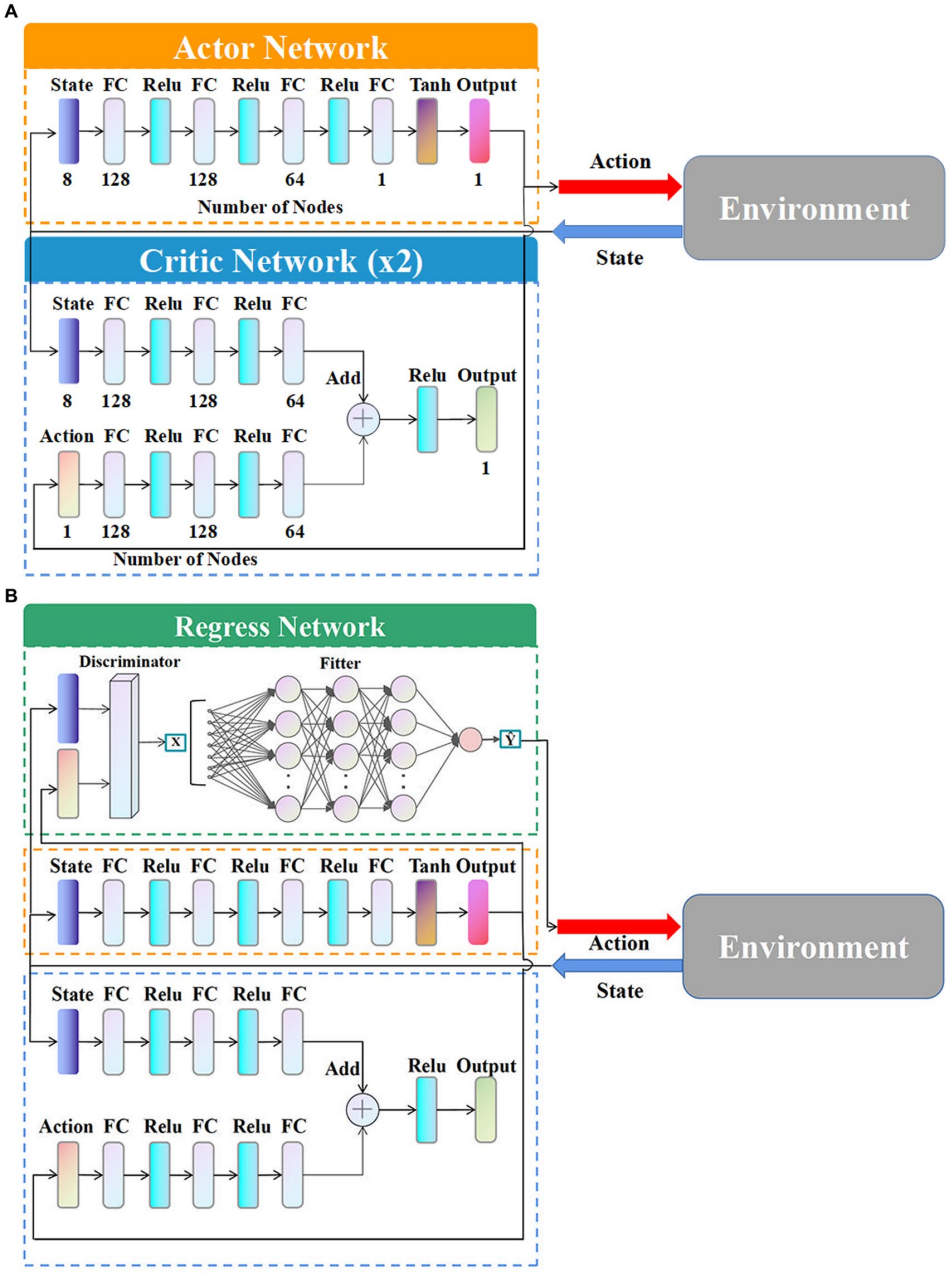
Deep neural network structure. **(A)** Network structure without regress network. **(B)** Network structure with regress network.

The deep neural network of the classical TD3 algorithm contains an Actor network and a Critic network, as shown in Figure 6A.

The types and numbers of layers in the algorithm are shown in Figure 6A. The numbers below the layers are the specific number of neurons.

The improved network structure with the regression network is shown in the Figure 6B.

As shown in Figure 6B, based on the original Actor network and Critic network, the regression network is newly added containing a discriminator and a fitter. During the agent training, the sample data, including the initial situations and maneuver instructions, generated from the interaction with the environment are inputted into the regression network as training samples. Those samples successfully evaded are filtered by the discriminator as $\hat{x}$ . And $\hat{x}$ are subsequently inputted into the fitter to train the neural network of the fitter. The trained fitter outputs the appropriate maneuvering commands $\hat{y}$ according to different situations instead of the original Actor network. The discriminator is based on the terminal equation (Eq. 10) to determine the evasion samples the successful evasion samples are set to 1 and the relevant information is inputted into the fitter, while the samples that fail to evade are set to 0 to be excluded. The fitter consists of multi-layer neural networks, which utilizes gradient descent to train the model and continuously adjust the weights and thresholds among the networks through backpropagation of errors.

Therefore, the regression network first screens the samples from the discriminator, and then, the selected samples are fitted based on the fitter. The overload of the regression network output is used as the whole output of the TD3 algorithm, which effectively improves the generalization of generating intelligent maneuvering strategies.

There are two reasons why the structure of the TD3 algorithm can be improved by introducing the regression network to enhance the generalizability. Firstly, it is susceptible to determine the HV’s success or failure in the hypersonic three-player pursuit-evasion game resulting in the easy design of the discriminator in the regression network. Secondly, the inputs and outputs of the hypersonic maneuver strategies are simple vectors, which do not require complex computation in follow-up processing. Because the hypersonic pursuit-evasion game itself is a highly dynamic process, the difference between different maneuver strategies is not significant and it is entirely possible to replace individual failure samples with successful evasion samples by fitting after screening, which in turn could improve the generalization of the algorithm.

In summary, the intelligent maneuver strategy proposed in this paper is based on the ITD3 algorithm, which utilizes the double training strategy to reduce the randomness of the initial training and improve the convergence of the TD3 algorithm, carefully designs the reward functions and sets the energy-saving factor to quantitatively regulate the off-target amount of terminals and energy consumption of process, and improves the network structure of the algorithm by introducing the regression network to improve the algorithm’s generalizability. The targeted improved strategy for the hypersonic three-player pursuit-evasion game can not only successful evade the cooperative interception under extended SPSD scenarios but also regulate energy consumption and have strong generalization.

## 4 Simulation

In this section, the effectiveness, generalization, and energy-saving of the intelligent maneuver strategy proposed in this paper are verified through numerical simulations. The relevant information used for the simulation validation is given in Section 4.1. The effectiveness of the proposed strategy under the expanded SPSD confrontation scenarios is verified by numerical simulation and comparison in Section 4.2, and the improvements at the level of generalization and energy saving are verified in Section 4.3.

### 4.1 Simulation information

The software selected for the simulation of this paper is MATLAB 2021a, and the hardware information is Intel (R) Core(TM) i5-10300H CPU @ 2.50 GHz, RTX 2060 14 GB, DDR4 16 GB, and 512 GB SSG. The parameter indexes used for the numerical simulation are shown in Table 1.

**Table 1:** Simulation, ITD3 algorithm training conditions.

Item	Value	Item	Value
March number $V_{E}/a,V_{P_{i}}/a$ (Mach)	6.0, 3.0, 3.0	Learning rate of Actor network and Critic network	0.005, 0.005
Maximum lateral overload $u_{\max},v_{\max i}$ (g)	3.0, 6.0, 6.0	Discount factor	0.9
Initial value of ballistic deviation angle $\psi_{vE0},\psi_{vP_{i}0}$ (deg)	0, pi, pi	Inertial factor	0.99
Initial relative distance $r_{EP_{1}},r_{EP_{2}}$ (km)	8.0, 10.0	Soft update rate	0.001
The lowest boundary value of miss distance $\delta_{1},\delta_{2}$ (m)	5, 5	The size of the experience pool	4000
The initial coordinate value of the typical situation 1 $\left(x_{E}\text{,}z_{E}\right),\left(x_{P_{i}}\text{,}z_{P_{i}}\right)$ (km)	(0, 0), (8, 0), (10, 0)	Sampling time	0.1
The initial coordinate value of the typical situation 2 $\left(x_{E}\text{,}z_{E}\right),\left(x_{P_{i}}\text{,}z_{P_{i}}\right)$ (km)	(0, 0), (8, 0.2), (10, −0.1)	Small batch sample size	128
The initial coordinate value of the typical situation 3 $\left(x_{E}\text{,}z_{E}\right),\left(x_{P_{i}}\text{,}z_{P_{i}}\right)$ (km)	(0, 0), (8, 0.05), (10, 0.03)	Evaluation round	5
Navigation coefficient $N_{1},N_{2}$	4, 4	Optimizer	Adam
Time constants of autopilot $\tau_{E},\tau_{P_{i}}$	0.5, 0.5, 0.5	Learning rate of regress network	0.01
Weight values of double training $w_{1},w_{2}$	0.99, 0.01	Target minimum error of regress network	0.001
Energy saving factor *E*	0.7	Minimum performance gradient of regress network	1*10^−6^

**Figure 7:**
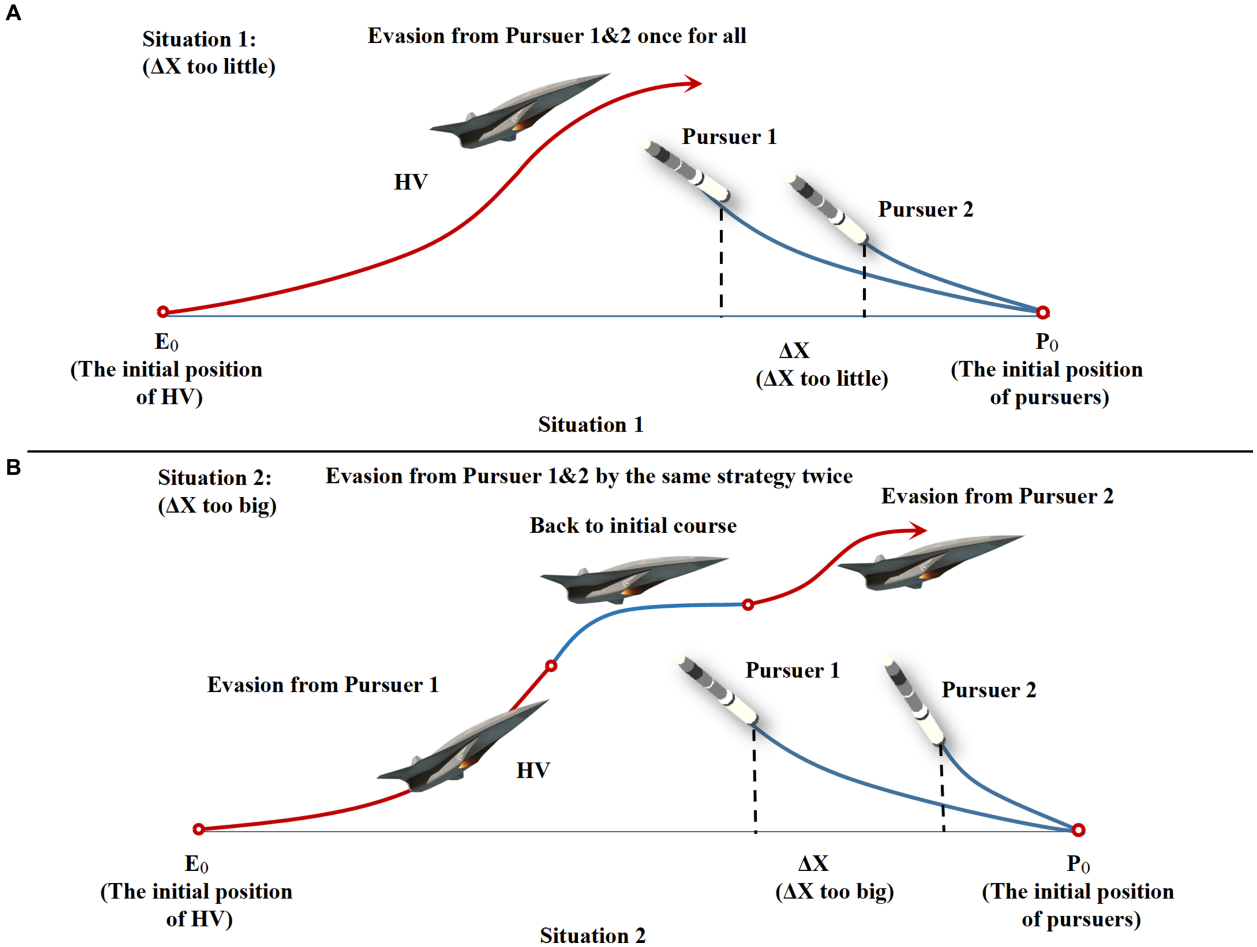
Evasion schematic diagram for situations 1, 2 of non-cooperative interception. **(A)** Space too little. **(B)** Space too big.

As shown in Table 1, the core performance indexes such as speed and overload of both pursuit-evasion sides all satisfy the characteristics of their respective vehicles. In the design of the critical off-target amount, which is the most important discriminating index for HV evasion, this paper sets it to 5 m based on the actual research anticipating the enemy strictly. And in the strict head-on situation, the relative distances between HV and the two interceptors are designed to be 8 km and 10 km, respectively, and 2 km for ΔX . The initial conditions of the two interceptors are carefully chosen, especially the spacing ΔX , which is too large or too small to achieve cooperative interception, as shown in Figure 7. Only when the interval is suitable, the HV must take into account the second interceptor when dodging the first one, and the complexity of a successful evasion will rise proportionately. The corresponding interception effectiveness is demonstrated when resisting against the classical maneuver strategy (Yan et al., 2020). In the subsequent simulations, the proposed intelligent maneuver strategy and the classical evasion strategy of the literature (Yan et al., 2020) are utilized to numerically simulate under the same three initial conditions in Table 1 to verify the improvement of the effectiveness of the proposed strategy. Generalization and energy saving effect are proved in comparison as well.

### 4.2 The validation of effectiveness

**Figure 8:**
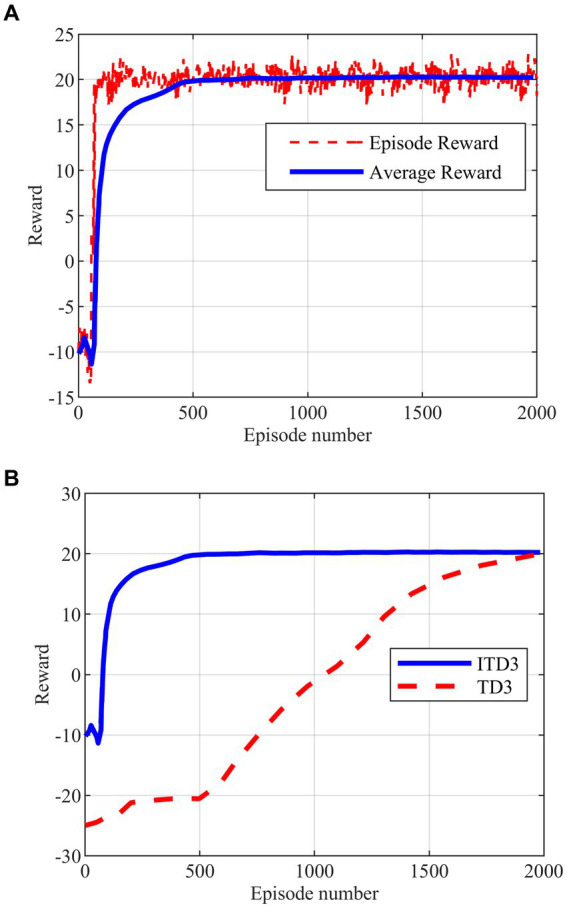
Agent training. **(A)** Improved TD3 training. **(B)** Comparison of training between improved TD3 and TD3.

The intelligent maneuver strategy proposed in this paper is generated based on the improved TD3 algorithm obtained by a variety of improved strategies. The maximum number of training rounds for the deep reinforcement learning algorithm is set to 2000 rounds, and the training process of the ITD3 algorithm and its comparison with the TD3 algorithm are shown in Figures 8A,B.

As shown in Figure 8A, the ITD3 algorithm can converge to the optimal solution in less than 500 rounds, while the TD3 algorithm starts to show the convergent tendency only in 2000 rounds in Figure 8B. The comparison shows that the double training strategy proposed for the algorithm training process is effective, and can well solve the problem of excessive randomness and high difficulty in agent training. The training stability and convergence of the algorithm in the hypersonic three-player pursuit-evasion problem are enhanced as well.

**Figure 9:**
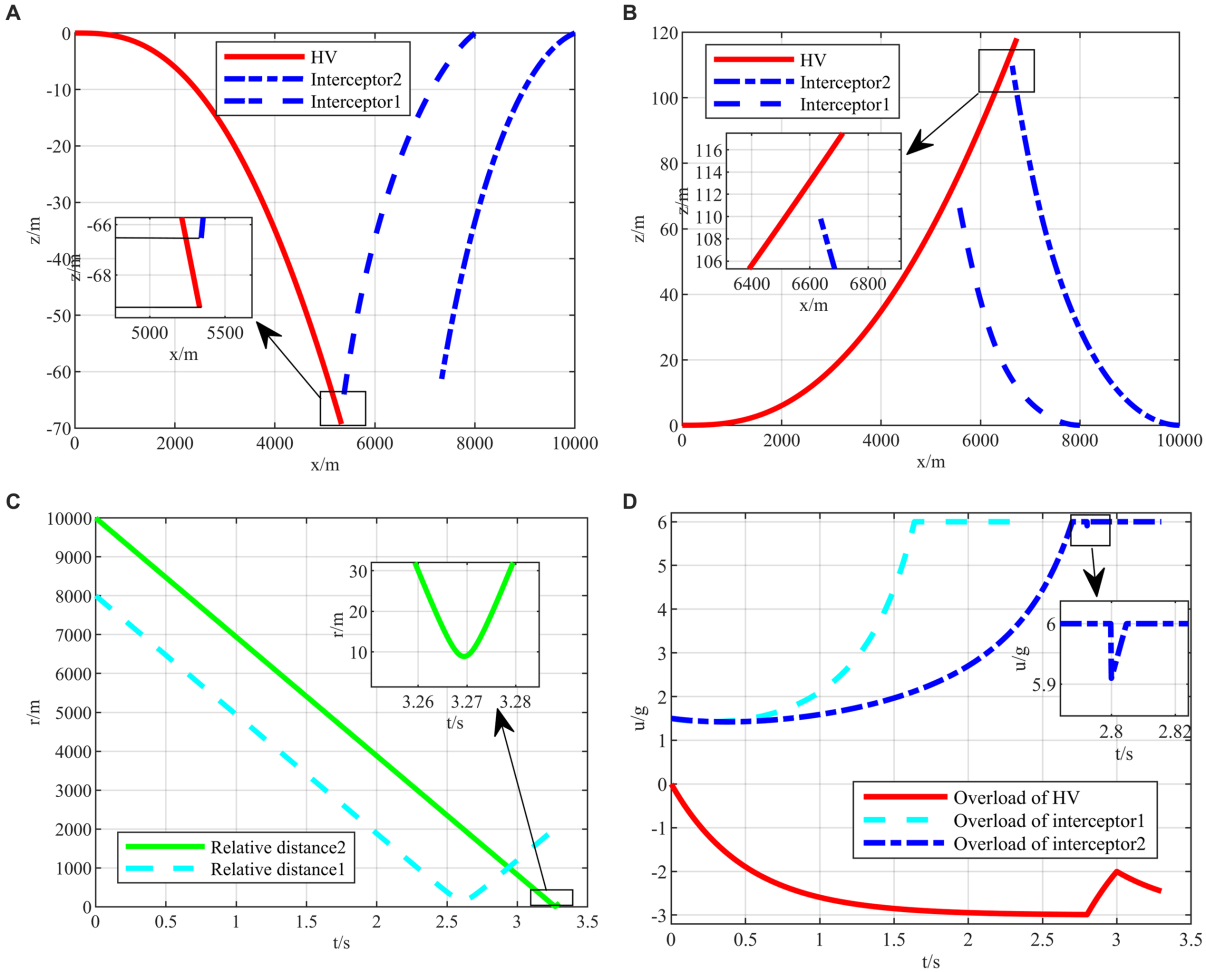
The HV pursuit-evasion game in the typical confrontation scenario 1. **(A)** Trajectory map under classical maneuver strategy. **(B)** Trajectory map under proposed strategy. **(C)** Relative distance under proposed strategy. **(D)** Overload change under proposed strategy.

**Figure 10:**
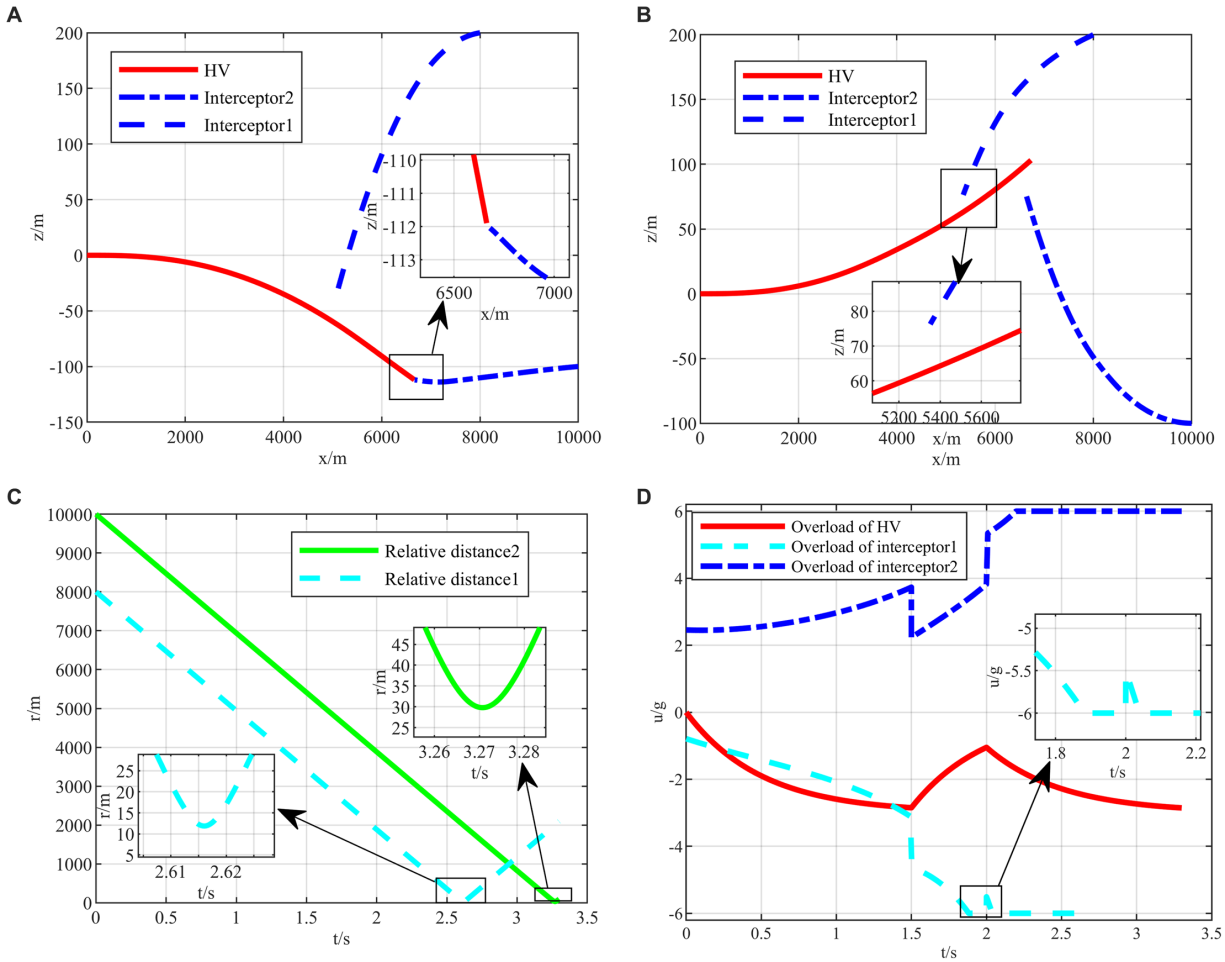
The HV pursuit-evasion game in the typical confrontation scenario 2. **(A)** Trajectory map under classical maneuver strategy. **(B)** Trajectory map under proposed strategy. **(C)** Relative distance under proposed strategy. **(D)** Overload change under proposed strategy.

**Figure 11:**
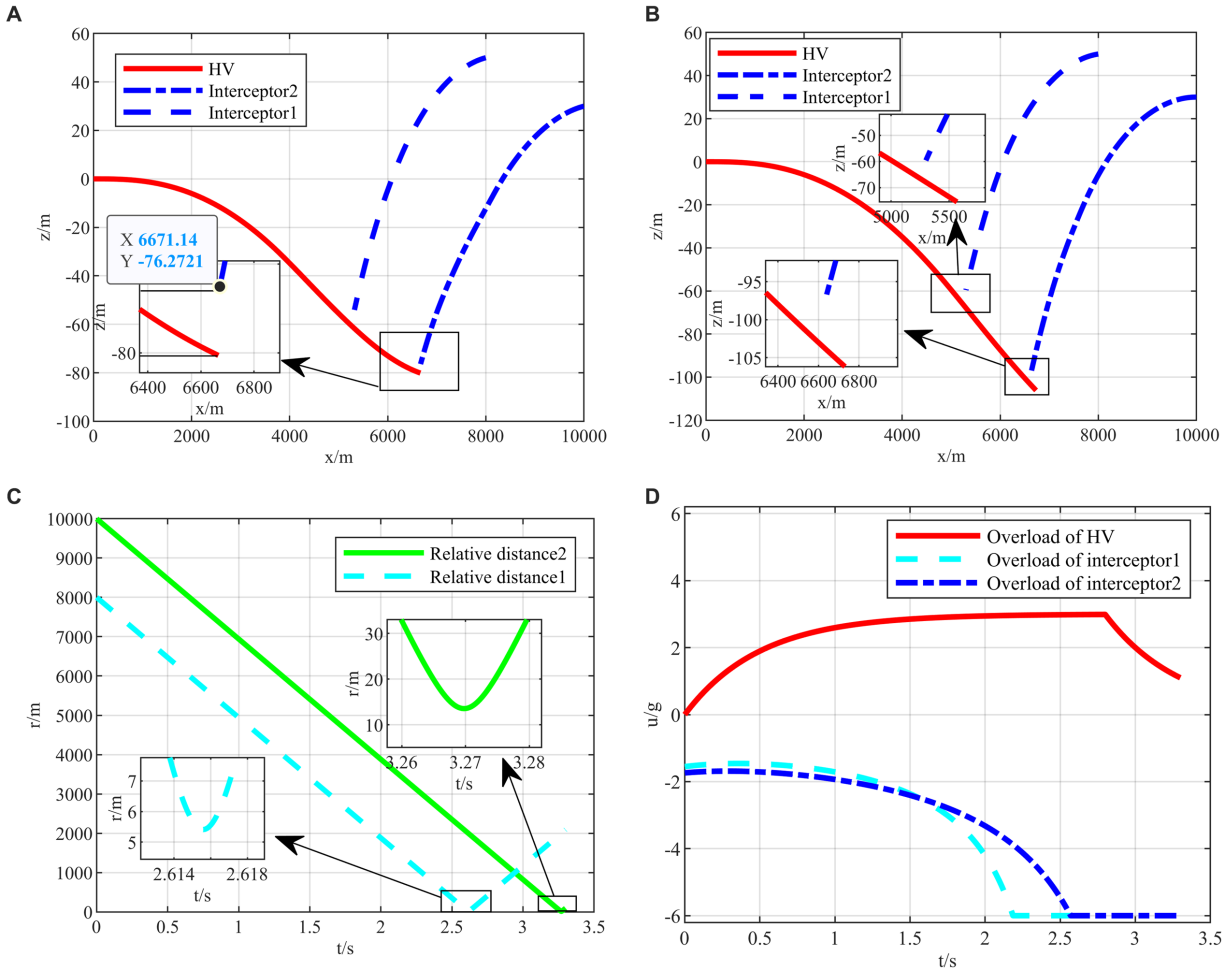
The HV pursuit-evasion game in the typical confrontation scenario 3. **(A)** Trajectory map under classical maneuver strategy. **(B)** Trajectory map under proposed strategy. **(C)** Relative distance under proposed strategy. **(D)** Overload change under proposed strategy.

After completing the agent training, the generated intelligent maneuvering strategy and the classical evasion strategy (Yan et al., 2020) are used to perform the simulation verification of the hypersonic three-player pursuit-evasion game under the above three typical confrontation situations, respectively. The simulation results are shown in Figures 9–11.

The HV encountering two interceptors in the typical confrontation situation 1 is shown in Figures 9A–D.

In the typical confrontation scenario 1, Figures 9A,B show the motion trajectories of the attacking and defending sides in a horizontal two-dimensional plane under the classic maneuver strategy and the proposed strategy, respectively. And Figures 9C,D demonstrate the relative distances and the overloads over time of aircraft under the proposed strategy. In other two subsequent confrontation scenarios, the meanings of the simulation diagrams are the same and will not be repeated.

As shown in Figure 9A, the terminal off-target amount of the classical evasion strategy facing the first interceptor is 2.2135 m, which is smaller than the critical miss distance, representing the evasion failure. On the contrary, the two off-target amounts of the proposed strategy are both larger than the critical miss distance, as shown in Figures 9B,C, representing that the strategy is successful under the strict head-on situation. It is easy to evade the first interceptor, and when facing the second interceptor, the strategy changes in the 2.8 s, instead of full overload maneuvering, leading to the relevant change in the interception guidance law of the second interceptor in Figure 9D.

The HV encountering two interceptors in the typical confrontation situation 2 is shown in Figures 10A–D.

In typical confrontation scenario 2, the classical maneuver strategy successfully evades the first interceptor but is intercepted by the second interceptor in Figure 10A. Meanwhile, the evasive commands generated by the intelligent maneuvering strategy are well-timed to achieve successful evasion of cooperative interception in Figure 10B. And both terminal off-target amounts met the requirements as shown in Figure 10C. It is worth mentioning that, unlike the classical maneuvering strategy, the intelligent maneuver strategy does not generate a downward overload command under the X-Z two-dimensional plane when facing the first interceptor above the *X*-axis, but rather, it drills through the two interceptors through the variation of overloading commands based on the upward maneuvering at the outset as shown in Figure 10D. It is different from the human’s expected maneuver instructions representing the intelligent algorithm’s abilities to explore and generate unexpected evasion strategies, which cannot be achieved by classic evasion strategies relying on the human’s subjective design.

The HV encountering two interceptors in the typical confrontation situation 3 is shown in Figures 11A–D.

In the typical confrontation scenario 3, the classical maneuver strategy is also intercepted by the second interceptor with the off-target amount of 3.5702 m smaller than the critical miss distance as shown in Figure 11A. When both interceptors are on one side to intercept HV, the intelligent maneuver strategy generates maneuver commands in the opposite direction in Figure 11B. In addition, when both interceptors are on one side, it is not easier to evade than the other two typical initial situations. The off-target amount of the first interceptor is only 5.545 m as shown in Figure 11C. In contrast, it is easier to avoid the second interceptor, and the overload can be appropriately lowered to save energy consumption in Figure 11D.

After numerical simulation and comparative analysis under three typical attack and defense situations, the effectiveness of the intelligent maneuver strategy in solving the hypersonic three-player pursuit-evasion problem is verified. Compared with the classical maneuver strategy, the proposed strategy performs better under difficult initial situations, which is more intelligent and effective in individual confrontation scenarios.

### 4.3 The validation of generalization and energy savings

**Figure 12:**
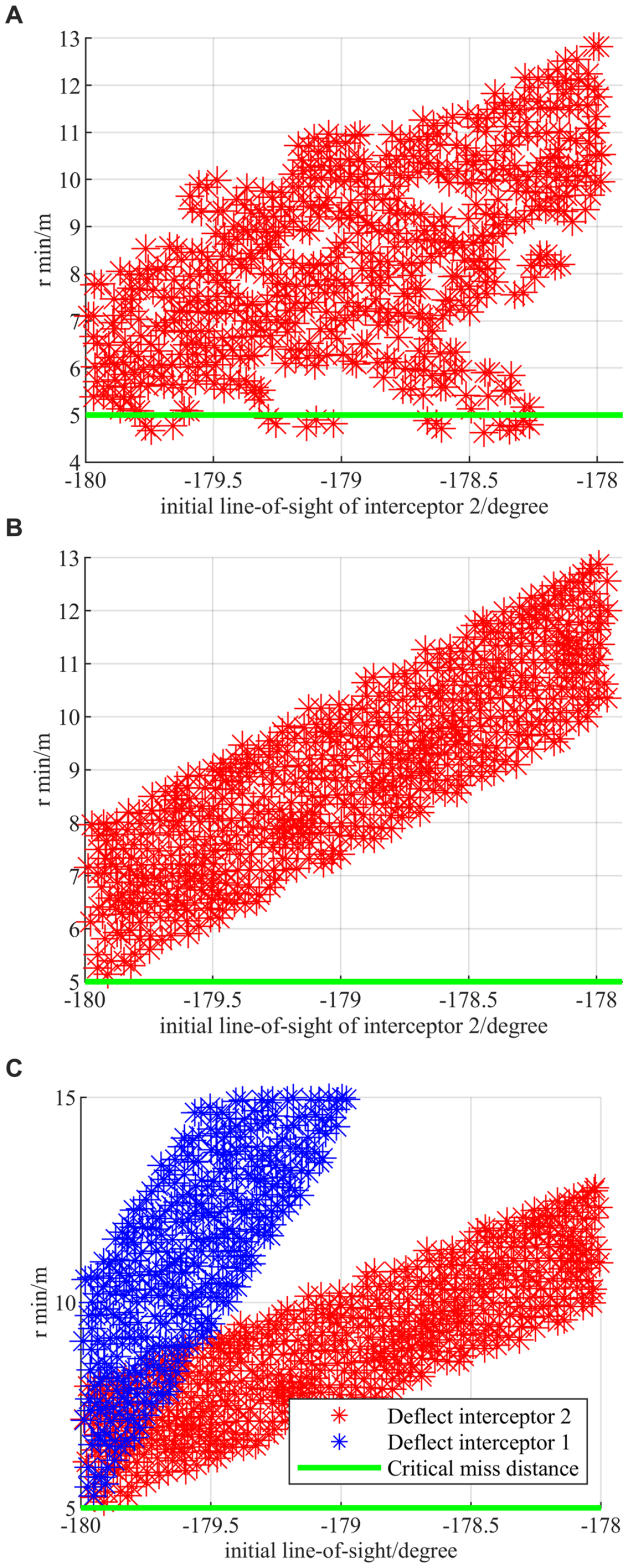
Monte Carlo simulations under the combined deflection. **(A)** Combined deflection without regress network. **(B)** Combined deflection with regress network. **(C)** Comparison between deflecting interceptor 1 and deflecting interceptor 2.

To further test the generalization performance of the proposed strategy, i.e., whether the regression network strategy is effective or not, Monte Carlo simulations are performed for the proposed strategy and the strategy without regression network, respectively. In Monte Carlo simulations, the initial parameters of two interceptors are, respectively, carried out the combined deflection, such as coordinates of x and z, ballistic deflection angles, and line of sight angles, based on the typical confrontation scenario 1. The simulation results are shown in Figures 12A–C.

The horizontal coordinate is the line-of-sight angle between the HV and the interceptor, and the vertical coordinate is the minimum relative distance during the evasion process. As shown in Figure 12A, there exist evasion failure samples where the terminal off-target amount is less than 5 m under the strategy without regression network. Figure 12B shows that the proposed strategy with the regression network can not only successfully evade in all cases but also show the linearity as a whole. Figure 12C demonstrates that the proposed strategy has excellent generalization regardless of which interceptor is pulled off and the evasion off-target amount for the first interceptor is larger than that for the second interceptor at the same deflection of the line-of-sight angle. It is proved through simulations that the generalizability of the algorithm can be effectively improved by the design of the regression network strategy.

**Figure 13:**
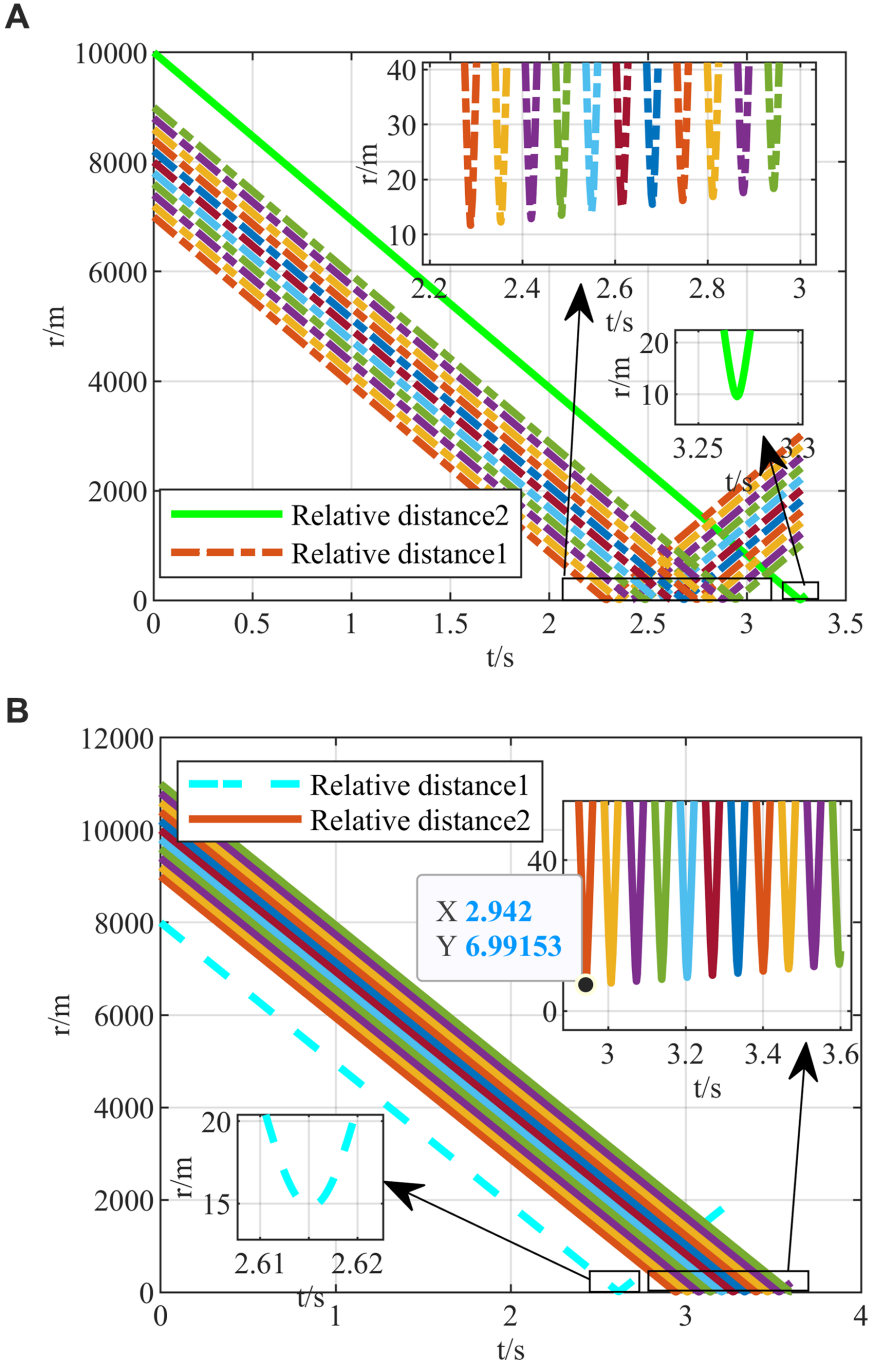
Monte Carlo simulations under the single deflection. **(A)** Single deflection of relative distance with interceptor 1. **(B)** Single deflection of relative distance with interceptor 2.

In addition to the line-of-sight angle, the initial transverse coordinates of the two interceptors are also individually polarized, and 22 game confrontation scenarios are generated. The terminal off-target amounts all satisfy the conditions of successful evasion, and their relative distances over time are shown in Figures 13A,B.

In summary, Monte Carlo simulations of various single and combined deflections demonstrate that the proposed strategy can achieve successful evasion in the face of different initial situations. The effectiveness of the regression network strategy is verified by numerical simulations.

**Figure 14:**
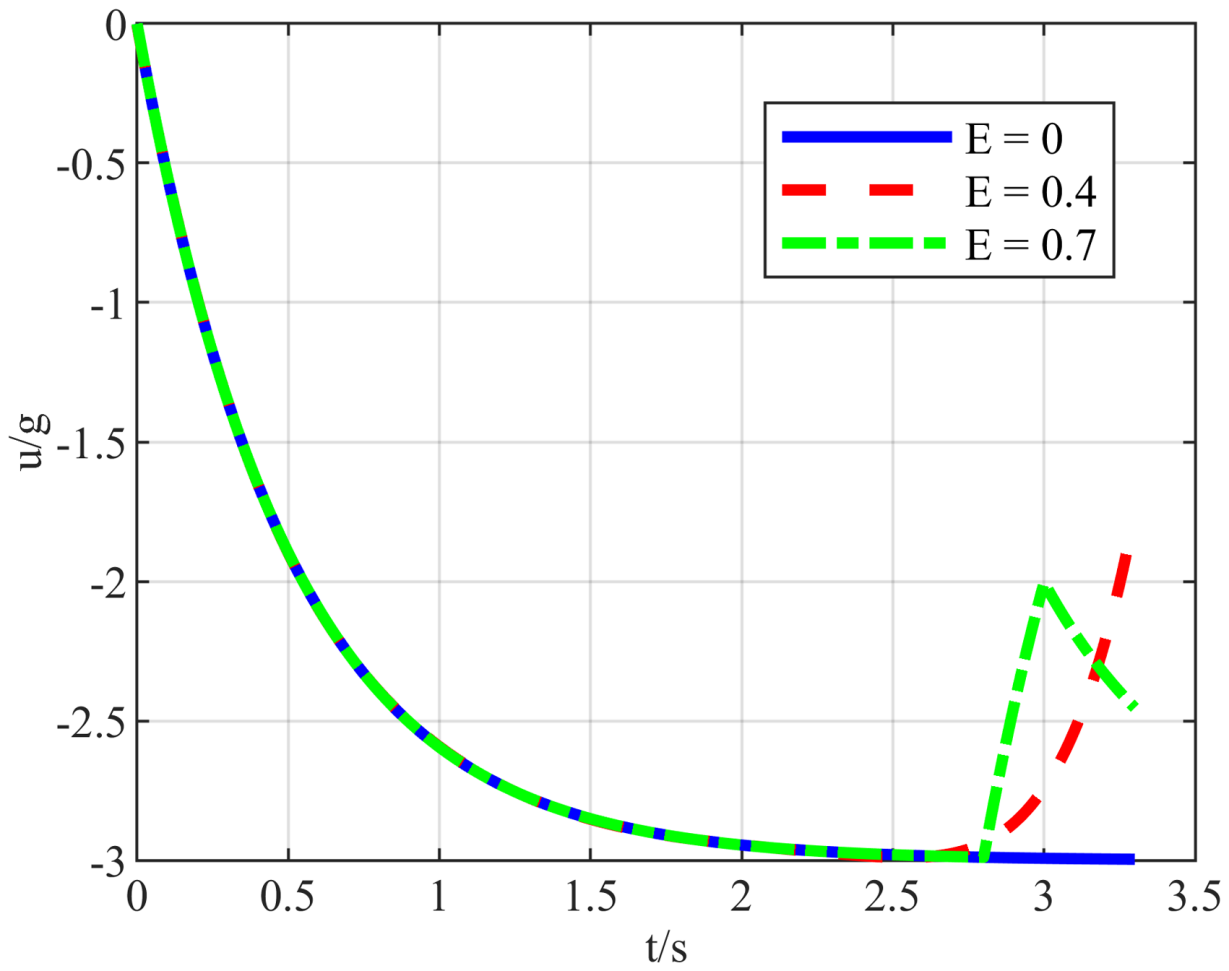
Variation of overload at different *E* values.

To further verify the improvement of the proposed strategy in quantitatively adjusting the HV energy consumption, ensuring that other factors remain unchanged and only the size of the energy-saving factor is adjusted, simulations are carried out under the typical confrontation scenario 1, as shown in Figure 14.

**Figure 15:**
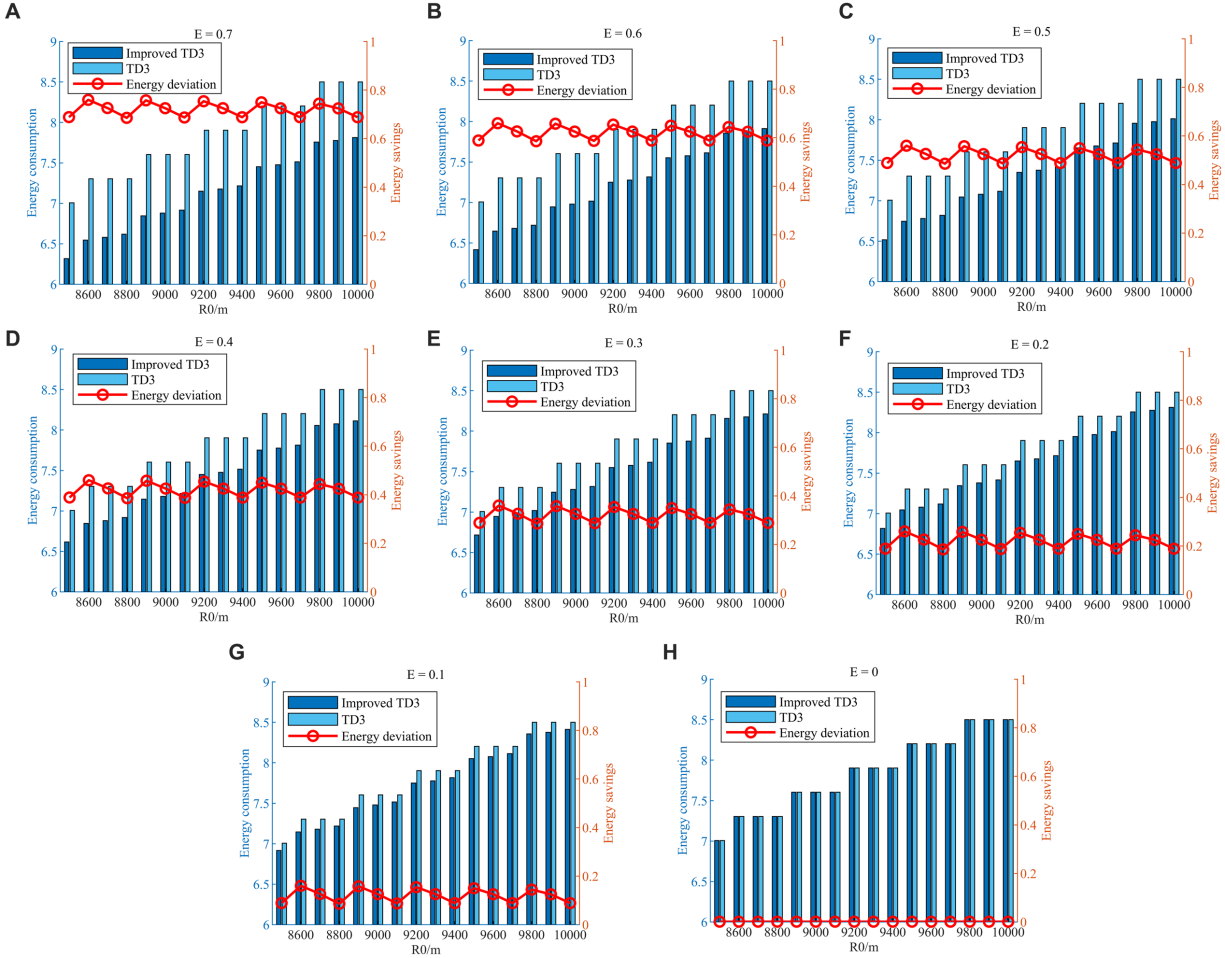
Energy consumption and energy savings with different *E* values. **(A)** *E* = 0.7; **(B)** *E* = 0.6; **(C)** *E* = 0.5; **(D)** *E* = 0.4; **(E)** *E* = 0.3; **(F)** *E* = 0.2; **(G)** *E* = 0.1; **(H)** *E* = 0.

In Figure 14, the maneuver overload of HV changes as the energy-saving factor is adjusted. When E=0 , HV chooses to maneuver with full overload without considering energy saving. And when E value increases, the integral of maneuvering overload in time is getting smaller under the premise of guaranteeing successful evasion. In addition, when E>0.7 , there is no guarantee that the HV can successfully evade two interceptors. Therefore, the energy consumption of HV maneuvering with and without the energy-saving factor strategy is calculated separately by varying its size when *E*∈[0,0.7]. The specific values of HV energy consumption at different E values are shown in Table 2, and the comparison simulations are schematically shown in Figures 15A–H.

**Table 2:** Energy consumption under different *E* value.

*E* = 0	*E* = 0.1	*E* = 0.2	*E* = 0.3	*E* = 0.4	*E* = 0.5	*E* = 0.6	*E* = 0.7
85017.34	84121.90	8346.65	82796.48	81026.42	79831.94	78049.78	76938.68

As shown in Figures 15A–H, the HV energy consumptions with different E values are normalized for plotting convenience, and the red connecting lines represent the energy saved, i.e., the difference in evasion energy consumption with and without the energy saving factor strategy. The energy saved during the evasion process likewise shows an overall increasing trend by continuously increasing the size of the E value from 0 to 0.7. From a separate figure, the energy saved at different initial relative distances fluctuates up and down around the E value. The above numerical simulations verify that the energy-saving factor strategy is effective, and the proposed strategy can quantitatively regulate energy consumption in the evasion process by adjusting the energy-saving factor E value.

## 5 Conclusion

In this paper, the intelligent maneuver strategy is designed to solve the three-player pursuit-evasion game problem, that a HV evades the cooperative interception of two interceptors. The expended SPSD scenarios are meticulously constructed to ensure the difficulty of HV evasion, and the proposed evasion strategy is generated from the improved TD3 algorithm, which is based on the TD3 algorithm and improved by double training strategy, energy-saving factor strategy and regression network strategy. The double training strategy considering two interceptors reduces the exploration blindness of the algorithm. By enhancing the deep neural network structure, the generalizability is improved by the regression network strategy. Starting from the reward functions, the energy-saving factor strategy achieves quantitative regulation of motorized energy consumption. Finally, numerical simulations are carried out to verify that the proposed strategy can achieve successful evasion in three typical confrontation situations of the extended SPSD scenarios where the classical maneuver strategy cannot achieve. Furthermore, the comparison analysis demonstrates the enhanced generalizability and quantitative energy saving capabilities of the suggested approach.

## Data availability statement

The original contributions presented in the study are included in the article/supplementary material, further inquiries can be directed to the corresponding author.

## Author contributions

TY: Conceptualization, Investigation, Writing – original draft, Writing – review & editing. ZJ: Conceptualization, Investigation, Methodology, Validation, Writing – original draft, Writing – review & editing. TL: Conceptualization, Software, Writing – original draft, Writing – review & editing. MG: Investigation, Writing – original draft, Writing – review & editing. CL: Formal analysis, Visualization, Writing – original draft, Writing – review & editing.
